# Bibliographical Notices

**Published:** 1851-12

**Authors:** 


### BIBLIOGRAPHICAL NOTICES.

*A system of Operative Surgery based upon the Practice of Surgeons in the United States, and comprising a Bibliographical Index and Historical Record of many of their Operations for a period of two hundred years.* By HENRY H. SMITH, M. D., &c. &c. *Illustrated by numerous steel plates—Parts* 1*st and* 2*nd.* (8vo. pp. 220 and 27 plates.) Philadelphia, 1851: Lippincott, Grambo & Co.

*Operative Surgery based on Normal and Pathological Anatomy.* By J. F. MALGAIGNE, Professeur Agrégé, &c. *Translated from the French by* FREDERICK BRITTAN, A. B., M. D., M. R. C. S. L. *Illustrated by wood engravings from designs by Dr. Westmacott.* Philadelphia, 1851: Blanchard & Lea.

In the first of the two works above announced, may be realized, to some extent, the fulfilment of a project that has long been entertained in various quarters, but never thought of, we suspect, as an undertaking on the scale in which it finally appears. In the second, many of our readers may discover the familiar features of an old and universal favorite of the European schools, which they will rejoice to find introduced at last among us in an excellent English dress. We wish them all success, and doubt not that they will both attain it, notwithstanding the number of reprints on similar subjects with which the country has latterly been favored. They are intended respectively to occupy distinct and different places, which have hitherto been vacant in our working libraries; and we trust they will be found to occupy them well enough to justify the enterprise of the gentlemen to whose liberality and care they owe the handsome style in which they are produced.

It would be hard to imagine a title more attractive to the practitioners and students of this country, than the one which Dr. Smith presents. Operative Surgery, so notoriously popular a theme with nearly all who are engaged in medical pursuits, is here offered in a guise, and under a prestige, which are admirably calculated to interest the feelings and meet the actual wants of the whole American profession. The beauty and extent of illustration with which, notwithstanding its convenient size and form and moderate cost, the author and his publishers have managed to enrich their offering, would amply suffice to command attention and place it in the front rank of standard publications; but its character as an essentially American work, built, as its title page imports, upon a strictly national foundation, is the one which must secure for it a general welcome, and for which we are especially glad to invite attention to it. No book has hitherto been written, which, in the language of the preface, "has presented the American practitioner with a comprehensive view of the opinions, operative methods and instruments of those of his countrymen who have given to American Surgery a character of its own." It has been the chief object of our author to fill this void within the smallest practicable compass, by means of condensed letter press descriptions and remarks, and very numerous accurate and beautiful engravings in the highest style of art.

This purpose of the author's appears to have limited somewhat the extent and fulness of his text, without, however, materially diminishing its practical utility. He "has not desired," we are informed, "to go over ground which has so recently been well displayed by writers both in Europe and this country. Many details of history, pathology, physiology, and surgical proceedings which are essential to a complete treatise upon the subject, have therefore been designedly omitted in this, as not coming within the scope of its plan."

Still in endeavoring to preserve the character of a manual of Cisatlantic operations, and basing his descriptions mainly on the views of his own countrymen, he does not allow himself to lose sight of the necessary Transatlantic aid, but has endeavored to display, in connection with others and his own, the opinion of "such European authorities as are universally received as sound." To these different descriptions he severally appends, in furtherance of his object as a teacher, an appreciation of the methods he describes, or an estimate of their advantages, which he wishes us distinctly to understand as "founded solely upon his own opinion," and therefore not to be confounded with the previous accounts of the various writers quoted.

But we must not regard his volume as a manual of operations only. He has materially increased its value and authority as a standard book of reference, in the curious choronological sketch of the progress of American Surgery, and in the still more interesting and instructive bibliographical index which appears in connexion with the different parts. This bibliographical index is a treasure in itself and a monument of the compiler's industry, which must win for him the gratitude and esteem of every medical inquirer who can appreciate the vast amount of labor which its preparation has required.

In the index are included, first, a general catalogue of native writers and publications on subjects connected with Operative Surgery, from 1783 to 1850 inclusive; secondly, a list of accessible American Journals containing papers on surgical subjects; and lastly, a list of distinct papers, to be found in the periodicals and elsewhere, on the topics of the different portions of the work. We find, for instance, under part 1st, bibliographical directions to sixteen different papers, "On Elementary Operations, &c.," and eight "On Etherization"; and under part 2d, two hundred and seven different papers are pointed out relating to the operations therein discussed.

These chronological and bibliographical records, and the large number of splendidly engraved and colored illustrations drawn from nature and from the best European models, constitute, in connection with its compact and very manageable form, the great features of the work, to which it will owe much of the favor which probably awaits it.

So much for its design and general character. We propose now briefly to examine into the arrangement of its letter press, and to note a few matters in detail, as they may present themselves in a necessarily rapid glance from page to page of the two parts before us.

The careful surgeon will be glad to learn from the opening paragraphs of his introduction, that our author is not disposed to place an undue value upon the mere manipulations of his art. "Appropriate constitutional measures," says he, "are therefore often as essential to the success of an operation as anatomical knowledge is necessary to its performance; and the happiest results will usually be obtained by those who closely attend not only to the local, but also to the general management of the cases on which they operate. For this reason, a successful surgeon must not only be a judicious practitioner of medicine, but also a devoted nurse and careful observer of the varying conditions of the system, under all circumstances. In every operation he should feel that he is largely indebted to nature; without her aid he can neither anticipate nor obtain success, whilst with it, especially as exhibited in the processes of adhesion or reproduction of tissue, he possesses a power that seems almost divine."

Pathological knowledge and tact, and ability in diagnosis, he rightly deems not less essential to success in operating, than the other qualifications just referred to; and hence he wishes his readers to remember that while, for purposes of methodical arrangement, "the ensuing pages are mainly limited to mechanical details, he nevertheless has no intention of giving them any other value than that of being one of the means of treatment occasionally demanded for the relief of disease."

With this explanation he proposes to divide the general subject into two leading parts: 1st, Minor Operative Surgery, or simply Minor Surgery; and 2d, Major Surgery, or Operative Surgery proper. As, however, he rejects the former of these, having previously made it the subject of a separate work, his object in the present instance, is, in a word, under the latter head, "to show the various modes of operating, and especially such as are resorted to by surgeons in the United States."

In accordance with this intention he arranges his work in five divisions. 1st, General Duties and Elementary Operations. 2nd, Operations on the Head and Face. 3rd, Operations on the Neck and Trunk. 4th, Operations on the Genito-Urinary Organs; and 5th, Operations on the Extremities." He adopts this regional distribution of his topics as that most apt to be suggested by the natural relation of parts. For a similar reason the details of each subject of inquiry are presented in the order in which the operator would be likely to attend to them. In the first place the anatomical relations would be thought of, next the methods of operating, then the instruments required, after these the dressings, and lastly the adjuvants necessary in the general or local subsidiary treatment.

The first Part, as it is entitled on the cover, is occupied with forty six pages of letter press in four chapters, with five plates of illustration. It is devoted to the consideration of 1st, the general duties of an operator; 2nd, Elementary operations; 3rd, means of arresting hemorrhage; and 4th, duties of a surgeon immediately after operating. Under the head of general duties of an operator, he begins with attentions to the patient, including the duties of a surgeon before, during and after an operation; next the care and management of instruments, including their action and selection, preparation and sharpening, manipulation and preservation; and lastly, the operator's duties to his assistants, such as their selection and instruction.

In discussing the duties of the surgeon before operating, he tells us that one of the most important is to save the patient unnecessary pain. This he advises to be effected through the agency of anæsthetics. He entertains no doubt of the safety of "Etherization," and does not hesitate to assert that "except in some few instances," (two of which he cites, by the by, with very little show of reason, so far as we can see, for the exception,) "the creation of pain by any operation can only be regarded at the present time as both unnecessary and injurious." And hence he naturally infers, that "philanthropy and that desire to ameliorate the sufferings of mankind, which is the true basis of sound practice, demand that neither prejudice, nor ignorance of its effects should longer prevent its employment by every operator." "In my hands," observes he again, "pure ether has been widely administered during the last four years; but for some months past, I have resorted to it mixed with chloroform, in the proportion of one part of the latter to five of the former, and I have yet to see the first patient in whom evil has undoubtedly resulted from its use." We have no time, nor do we feel inclined, to dwell upon the anæsthetic question. We only quote our author in relation to it now, in order to show the decided ground he takes in favor of the practice; and at the same time to express the gratification we feel at finding it so earnestly and frankly recommended in a treatise, which, being avowedly put forward as a sort of mirror of our national operative science, must be looked upon by many at a distance, as at least a fair exponent of the Philadelphia creed. Happily the conclusion, this time at all events, would prove a just one, in spite of the extremely conservative resistance to the prevailing current of opinion and experience, which still exists in certain influential quarters of this city.

Under the caption of duties after the operation, we find among other matters very briefly touched upon, some general hints respecting the kind of diet proper at this period, and especially suggesting the policy of avoiding in many cases a sudden change or excessive reduction in the amount and character of nourishment. His ideas on this matter might have been more clearly given, yet as they contain nothing particularly new, we allude to them only for the purpose of protesting against the impression likely to be conveyed by the language of a portion of the concluding paragraph, as follows: "A routine practice in any complaint," says he, "is always bad." No one will venture to deny this venerable axiom; but with the remainder of the sentence the case is very different. We have it in the following words:—"and in this respect American surgeons have perhaps followed too closely the precepts of their European brethren, forgetful of the different habits of our people, and of the daily developments of science."

He further adds, "meat, and that often in large quantities, is the daily food of all the laboring classes, as well as of many others, in the United States, whilst thin soup, salad, and wine is the diet of most of the same class on the continent. Low diet, therefore, is not so great a change to them as it would be to patients in this country, and the American surgeon may, in many instances, advantageously pursue a practice more in accordance with his own locality."

Now we are not willing to admit any one of the affirmatives that are thus jumbled up together in the sweeping, but no doubt unintentional reflection on American surgeons and "their European brethren" contained above. We do not believe—indeed the pages of the book itself most honorably contradict it—that American surgeons, (we mean those of mature age and experience, and who have been trained at home,) are "forgetful of the different habits of our people, and of the daily developments of science"; nor is there any reason to suppose them in the habit of following the precepts of any single set of men in a matter of daily observation in which they are quite as well prepared and competent to form their own opinion as the oldest of their brethren, far or near. Lastly, we were not before aware that European operators were addicted to a routine practice, no matter in what class; or at any rate, that their "precepts" were not intended to apply to as great a variety of classes and accidental circumstances as well as operations, as had become familiar to us here. True it is that the young men who go abroad to walk the hospitals, and too readily content themselves with a crude experience that is much oftener the evidence of things not seen than the substance of things hoped for, have few opportunities of watching the treatment or results of operations on any classes similar, physically, morally or in previous habits, to the parties who become the subject of the knife at home. But it is idle to consider the occasional acts and dicta of these junior observers as the expression of the scientific habit of the land. The therapeutic precepts and practice of authoritative surgeons of this country, were long since established on the same broad foundation of general principles which supports the philosophy of surgery throughout the world, whether it deal with the prince, the burgher, or the peasant, the rich or the poor, the employed or unemployed, of whatever kindred, tongue or people. These principles have come down to us *in verba magistri* from both sides of the Atlantic; changeful and changing only through the media by which they may be studied, and in their great outlines so marked and clear that he who runs may read them.

Chapter 2nd is devoted to Elementary Operations, including Incisions, Dissections, and Punctures.

Chapter 3rd treats of the means of arresting Hemorrhage, such as Compressions, Ligatures, Styptics, Cauteries and other usual expedients.

Chapter 4th, under the head of Duties of a Surgeon immediately after Operating, presents us with some account of dressings, of the closing of parts after an operation, and of the means employed to favor union.

In regard to dressings, and the great importance of attention to them, together with the frequent indifference of students to this stage of operations, we find some very just remarks. "The first and subsequent dressings of an operator are the real tests of his surgical skill. In making them, he first proves his claims to the high position of a surgeon, and rises above the grade of the 'cutter.' Before this the surgeon was limited to the mechanical portion of his profession, but in the dressing and after-treatment he has an opportunity of showing his judgment and the resources of his science." It is a matter of regret that while upon a topic considered so important, a few more precise and definite instructions were not given in this place, in addition to the special directions which are reserved for after consideration in the study of each operation. We would be glad to know, for instance, the usual precautions to be taken in the removal and renewal of the first and subsequent dressings just spoken of, and how soon and how often, as a general rule, these dressings ought to be applied. A similar lack of definite information may be observed, we think, in regard to minor points throughout this chapter. Thus in reference to means of retaining parts in apposition, isinglass receives no notice, collodion is barely spoken of, and the only adhesive strip really described, is directed to be about half an inch in width.

The first plate of the illustrations of the work, gives accurate and beautifully finished side views of some of the instruments employed in making incisions and dissections, in the extirpation of tumors and the ligatures of arteries. Plate 2nd, represents various instruments employed for the arrest of hemorrhage, extraction of polypi, &c. Plate 3rd, presents a beautiful series of delineations, modified and drawn from Bernard and Huette, of the six positions of the scalpel. In plate 4th, we have nine figures relating variously to the arrest of hemorrhage by the compression and ligature of arteries. The fifth and last plate of the first part exhibits in seven capital figures a front view of various incisions, and of the different modes of uniting wounds by sutures or adhesive plasters.

So large an amount of space has been already occupied with the notice of the introductory portion of the work of Dr. Smith, that we will be forced to pass much more hurriedly over what remains. This is the less to be regretted, inasmuch as it would be impossible to do any justice to the specialities and various details of so many and such important processes. All that we can pretend to do, must be to present a rapid sketch of the general order of arrangement, and an enumeration of the different sets of operations figured and described, and to accompany them in passing, with a few notes here and there in connection with certain points of interest.

Part second, then is occupied, as a whole, with operations on the head and face, and opens in the first chapter with the surgical anatomy of the head.

Operations on the head are treated of in chapter 2nd in two sections, the first being on operations on the scalp; and the second, on operations upon the bones of the cranium. They are illustrated in three plates, showing the instruments used upon the bones of the head, certain operations on the scalp and bones of the head, and thirdly, a side view of the structures of the head and of the operation of trepanning. Among operations on the scalp, we have the extirpation of encysted tumors, nævi materni, cephalœmatoma, and division of the supra-orbitar nerve. Then for the operations on the bones of the head, those for caries, necrosis and exostosis of the cranium are briefly sketched, also trephining the cranium, puncturing the head for hydrocephalus, and the removal of fungoid tumors of the dura mater.

Various operative expedients are described for the obliteration or removal of nævi materni or erectile tumors. Of the different methods of removal, Dr. Smith prefers excision, where the skin is not much involved in the disease, or the tumor larger than a walnut ; the hemorrhage being in most instances, according to his experience, readily controlled by ligature or by pressure, particularly when care is taken to incise only the healthy structure, and not to open the tumor. In case of larger size of tumor, in which the hemorrhage is more to be apprehended from the ordinary extirpation, he recommends the operation of Dr.

Physick and Dr. Wm. Gibson, by partial incisions renewed at intervals, the vessels being taken up as they are divided, and the edges of the wound being prevented from uniting by the interposition of lint between their opposing surfaces. We doubt the propriety of advocating the excision of these tumors as an easy and safe operation, in preference to the various expedients which are unattended with the loss of blood. The operation with the knife, no matter how performed, is unquestionably a difficult and dangerous one. In the hands of eminently skilful and dexterous surgeons, on more than one occasion, it has proved positively frightful, so much so as to have led some of the first operators of their day to reject it altogether; and it ought not, in a work intended for the guidance of students and junior practitioners, to be proposed, without a special warning of the danger that might possibly ensue from its performance.

Although exostosis of the cranium is alluded to and even represented on a plate, nothing is said of spina ventosa of the skull. Here was a chance of introducing Dr. G. McClellan's case (quoted, with a portrait, by Dr. J. H. B. McClellan in his edition of his father's Surgery, p. 348,) which we regret to find neglected. As a rare and instructive operation by a noted Philadelphia surgeon, it was at least as worthy of special record as that of Dr. Warren for tumors of the dura mater.

Passing over the remaining pages of chapter 2nd, in which appears an excellent account of the operation for trephining, we come to chapter 3rd, containing a brief view of the surgical anatomy of the face; and to chapter 4th, which treats of operations practised on the eye-lids and on the lachrymal apparatus. Among those upon the eye-lids, we find many extremely interesting autoplastic devices for the relief of various deformities, all of which are well described and represented.

Next comes chapter 5th, on operations on the eye-ball, including those upon the external coats and on the muscles of the ball, as well as on the globe itself. This series, of course, introduces the subject of strabismus, the different methods of operating for which are carefully figured and explained.

In regard to the extirpation of the eye-ball, for which the "ordinary operation" is given very cursorily in the same section with strabismus, we note a direction for arresting the hemorrhage "by stuffing the orbit with lint." This is not in accordance with the principles inculcated in some other portions of the work, and is certainly not according to the best authority. The practice is allowed by continental surgeons, although Malgaigne recommends only small pellets or plugs of lint to stop the bleeding; and Sedillot, while directing the use of charpie to fill the orbit, expressly says, there is nothing to fear from hemorrhage. It is discountenanced, however, by British operators, among whom we may refer to Lawrence and Skey. The former objects to it, for good reasons which he gives in full, and the latter calls it "a highly dangerous proceeding."

After these we meet in chapter 6th with the anatomy of the eye-ball, and of the iris in its peculiar relations, the operations for cataract, and those for the formation of an artificial pupil.

Several matters of interest present themselves in the course of these short sections on ophthalmic operations; particularly in relation to extraction, the removal of strabismus, and the various modes of producing artificial pupil, including the operation of our townsman, Dr. Hays. The limits of our notice, however, oblige us to hurry by all of these, and, indeed, to pass over in the same superficial manner the seven extremely interesting chapters which complete the volume. Chapters X, XI and XII especially are rich in records of the triumphs of American Surgery, and in details which are in the highest degree valuable and attractive; and we would gladly spend an hour, did time allow, in culling from their pages many hints suggested, and curious facts that might be noted in connection with the various admirable autoplastic and resection operations, in which the dexterous ingenuity and sagacious enterprise of our countrymen are seen to make a characteristic impress on the extension of this province of the healing art. We must content ourselves, however, by stating in very few words that the synopsis of operations on the eye is followed by a short account of metoplasty, as exemplified in a case of Dr. Watson's of New York; by a chapter (VIII) on the external nose, including rhinoplasty; by another chapter (IX) on the internal nose; by a full and very interesting succession of chapters (X, XI and XII) on the mouth, externally and internally, along with tongue and throat, embracing among other topics, hare lip, cheiloplasty, genioplasty, and salivary fistula; on resections of the upper jaw bone and lower jaw-bone, and on staphyloraphy and staphyloplasty. Finally, we have in chapter XIII the concluding sections of the published portions of the work. They are devoted to the demonstration of the operations on the ear. Each of these chapters, it must not be forgotten, is furnished with its section on the appropriate surgical anatomy, and also with its splendid steel engravings. Several of these latter, we observe, are after Bernard and Huette, Bourgery and Jacob, Liston and other British and continental sources; but a considerable number, and some of the very best, are original, or from American authorities, and all may be regarded as unsurpassed in their peculiar style by those of any publication in the Union. We shall look with great pleasure for the concluding portion of the work, which we hope to be able to notice more in detail than has been possible on this occasion; meanwhile the author, publisher and artist will accept our heartfelt thanks, in behalf of all who take any interest in our opinion of the enterprise, for the very handsome manner in which it has been thus far carried out. We sincerely hope their efforts will meet with the prompt and ample recompense which, in these days of reprints and second hand translations, they have a right to claim for the benefit of native letters, if not for the good of their native land. No American practitioner who has this good at heart, should neglect to provide himself with an early copy for his table, not only as an instructor that may often prove a friend in need, but as a noble evidence of better times in prospect for the home teachers of the *lex scripta* of his art.

But little room remains for us to dwell upon the translation of Malgaigne. Indeed, the reputation of the work, whether French or English, is by this time so entirely established, that very few words will enable us to say all that is necessary for the information of our readers. For years past this manual has been the ruling vade mecum of the students of all the European schools, and has been largely read and quoted from in Great Britain and America. We venture to aver that there has not been in Paris for the last ten years a student of surgery from any country, who has not diligently conned and thumbed its well known pages. Yet, strangely enough, it had passed through four large Parisian editions, one Belgian edition and five translations, before it found its way across the channel, except in its old Parisian shape. And, still more strangely, five years more have been allowed to pass before any one has thought of republishing its English counterpart for the benefit of the American profession.

It must prove an acceptable addition to our stock of aids and comforts of this kind, even in the midst of many other similar productions, because it is beyond comparison the most complete and comprehensive as well as most compact; while for clearness, accuracy and methodical arrangement, it is no less superior to all the manuals extant. We speak of it as a manual of operations only. More than this is not intended by its author. His views of what ought to be required in a full treatise on Operative Surgery are vastly more extended. The limits of his work restricted him nearly altogether to the surgical anatomy and the different operative manipulations. These have, however, been treated with so much special care and with such variety and fulness, as well as precision, of detail and description that his volume may safely be regarded as an indispensable adjunct to the less systematic and comprehensive publications of Liston, Fergusson, Skey, and others, which have been reprinted or otherwise issued in America. We say nothing of the larger historical and illustrated works of Velpeau, Pancoast, Smith and others, since they are manifestly of a different character.

The work is divided into three parts; the first being upon elementary operations, the second on general operations and the third on special operations. These three grand divisions embrace (with the exception of the use of bandages and other dressings, and the management of fractures and luxations, purposely omitted) the whole range of manipulative surgery. His plan in describing an operation is to begin with a concise but lucid sketch of the surgical anatomy of the part concerned; then to proceed with a methodical and minute account of the different stages of the ordinary process, in regular order of succession; next, a history of the various important modifications that may have been adopted or proposed; and lastly, to submit a comparative appreciation of the different methods just described, together with a view of their probable effects.

Mr. Brittan is entitled to our thanks, not only for the fidelity and elegance of style with which he has discharged his obligations as translator, but for the numerous additions which he has appended in the form of foot notes, relating chiefly to British operations or improvements of later date than the authors' last edition. The value of the book is still more decidedly increased by the handsome and well selected illustrations from the drawings of Dr. Westmacott. These wood cuts and the English notes have rendered the American reprint actually more desirable than the original work; and nothing is wanting but a chapter on the present modes of blunting the sense of pain—on anæsthetic inhalations, in other words—to make it all that need be looked for as a model of its class.

---

*Outlines of Chemistry for the use of Students.* By WILLIAM GREGORY, M. D., Professor of Chemistry in the University of Edinburgh. *First American from the Second London Edition, Revised, corrected and enlarged.* By J. MILTON SANDERS, M. D., LL. D., Professor of Chemistry, Pharmacy and Toxicology, in the E. Medical Institute of Cincinnati, &c. Cincinnati, H. W. Darby & Co., 1851. pp. 614.

This work of Dr. Gregory sustains his reputation as a teacher of Chemistry, and confirms the favorable opinions, formed from his previous labors in the same department of science and in conjunction with Professor Liebig. The selection and discussion of the facts and theories, evince judgment and discrimination in their adaptation to the wants of the student, and this arrangement is in general conformity with the plans adopted by some of the British Chemists, following in this respect more especially, in the inorganic part, the work of Turner. One alteration from this general plan, the entire omission of the class of imponderables, is striking, and based on correct principles, and no doubt was adopted with due attention to the requirements of those students for whom the work was intended. We cannot, however, consider it as adapted to the present position of students in the United States. These subjects are in many parts of essential importance, especially to the medical student; and although it is very desirable that he should be thoroughly grounded therein, previous to commencing his medical studies, yet, in the great majority, we find an almost total want of this knowledge, the continuance of which deficiency would be a material obstacle to his future progress in chemical knowledge. The space which has been obtained by this omission, has been usefully devoted to the extension of the organic division of the work. It would have been desirable that a work emanating from a chemist of such high reputation as Dr. Gregory, should have been written with great clearness and perspicuity, especially as intended for those to whom the subjects are, if not entirely new, certainly not familiar. Students generally consider chemistry as a difficult branch of science, and hence require the instruction to be conveyed in such form, that the ideas may be received without any revision of the language used.

We regret that we cannot speak in the same favorable terms of the revisions and corrections of the American edition. More reflection should have been bestowed on some of the few foot notes which have been appended to the original work, for we cannot attribute to any other cause, the singular assertion on page 75, that lime owes its greater degree of solubility in cold water, to the greater quantity of carbonic acid in cold than in hot water. At page 474, a claim is laid for the discovery by Professor King of "resinoids" in several indigenous plants, as phodophyllum, cimicifuga, &c., thus conflicting with similar claims made by W. S. Merrill, A. M., in July 1850. Whatever priority either of these gentlemen have, as to some of those substances mentioned, neither can lay even the shadow of a claim to the very first on on the list. Students have been made aware, through the pages of a work in all their hands, (United States Dispensatory,) of the existence in the root of May-apple of an active bitter principle, called podophyllin, the discovery of which dates as far back as 1832, as reference to the Journal of the College of Pharmacy, Vol. III., p. 273 will show; and in addition, the same Journal for 1847, (vol. xix. p. 165,) contains a detailed analysis, in which the activity of the drug as a drastic cathartic is traced to two resins, one of which was obtained pure and colorless.

The American Edition, it is stated, "is more perfect than the London one; numerous errors, which had escaped the author, being corrected." It is to be regretted that this has not been carried to a greater extent, those remaining being sufficiently numerous. It is only necessary to cite a few. The formulæ for the three compounds of phosphorus and hydrogen, as given on pages 132 and 134, differ, and three out of the six are incorrect. P. 177, magnesia "is best obtained pure by heating the carbon to redness," meaning carbonate. Sweet oil of wine is $2S_3O + AeO + C_4H_4$" the 3 in the sulphuric acid being transposed &c. We should have supposed that the isolation by Frankland, of compounds, having an identity of composition with ethyle and amyle deserved a passing notice, from their relation to the theory of compound radicals, even if the Editor did not confide in the results. Another point of practical importance struck us in relation to an assertion at p. 188, in connection with the hydrated sesquioxide of iron. "In its moist state it is the only known antidote to arsenious acid." The efficacy of magnesia, in the same cases, has now so much testimony in its favor, that the above unqualified assertion is decidedly objectionable, and however true at the time of original publication in 1847, requires correction by means of the knowledge subsequently obtained.

In typographical execution, paper, &c., the work can fairly compete with its congeners of the Eastern States.

